# Multifunctional Lysyl Oxidases

**DOI:** 10.3390/ijms24076044

**Published:** 2023-03-23

**Authors:** Philip C. Trackman

**Affiliations:** The Forsyth Institute, 245 First Street, Cambridge, MA 02142, USA; ptrackman@forsyth.org

This Special Issue on lysyl oxidases, which are proteins derived from five related genes known as *Lox*, and *Loxl1*–*Loxl4*, brings together articles that reflect some of the diverse approaches and perspectives needed to better understand the biology of these multifunctional proteins. Lysyl oxidases are multifunctional proteins involved in the biosynthesis and maturation of elastin and collagen and have additional functions. Each paralogue has roles in developmental biology, extracellular matrix production, tumor biology, fibrotic diseases, while various extracellular and intracellular mechanisms and activities of the enzymes and proteolytic fragments derived from their pro-enzymes occur. The articles in this volume touch on all of these topics. Specifically, articles cover the investigation of the three-dimensional structure of LOXL2 [1,2,3]. Importantly, the effectiveness of a novel and remarkably well-tolerated inhibitor of lysyl oxidases developed by Pharmaxis Corp of Australia is highlighted as being highly effective in fibrotic conditions in preclinical studies [4]. The inhibitor is now being used in human clinical trials to address myelofibrosis. The developmental importance of *Loxl2* and *Loxl3* genes is demonstrated in the perinatal lethality in *Loxl3* knockout mice with partial penetrance and partly overlapping functions with *Loxl2*, involving skeletal development. Surviving *Loxl3* knockout mice exhibited a smaller size and had a shorter-than-normal lifespan. Double *Loxl2*/*Loxl3* knockouts showed early embryonic lethality, while global overexpression of Loxl2 protein in the *Loxl3* knockouts partially compensated for *Loxl3* knockout, clearly supporting the distinct yet overlapping roles of the two proteins in skeletal development and overall survival [5]. It is, therefore, apparent that lysyl oxidase paralogues, while being distinct entities with unique functions and expression patterns, must work in concert to accomplish normal development and longevity.

The fact that LOX paralogues could have specific regulatory functions independent of catalytic activity is highlighted in an in vitro study in which all five paralogues were sequentially knocked out in the human breast cancer cell line MDA-MB-231, followed by re-expression of LOX and other paralogues. Of the differentially expressed genes, the expression of only four genes was strongly dependent on LOX re-expression and not dependent on the other paralogues’ re-expression [6]. This presents the notion that LOX paralogues could have unique regulatory functions despite the conserved sequence of the catalytic domains. The regulatory activity was further shown to be independent of the enzyme activity of LOX [6]. Thus, paralogue-specific non-enzymatic regulatory functions of LOX (and probably other paralogues) are implicated by this work. This concept is consistent with our work and the work of others showing that the lysyl oxidase propeptide, which has no lysyl oxidase enzyme activity, has biological activity, as also reviewed in this issue [7].

In recent years, new proteolytic processing sites separate from the known procollagen C-proteinase/BMP-1/TLL-1/2 site, plus additional post-translational modifications in LOX and LOXL1, have been identified by Rodriquez-Pascual and colleagues [8,9]. In this issue, LOX was shown to have an additional processing site for ADAMTS2 and 14 in addition to the known BMP-1/TLL site pointing to undiscovered potential functions of the released peptides, and/or the enzyme isoforms generated. Post-translational sulfation of LOX was also shown to potentially have consequences for LOX activation and collagen binding [6]. New structural insights into the three-dimensional structure of LOXL2 in papers by Minae Mure and colleagues provide important information regarding the catalytic mechanism of action of LOXL2 and LOX based on molecular modeling analyses [3]. A second paper presents the development and likely geometry around disulfides in LOXL2, as determined in detailed mass spectrometry analyses of preparations of highly active recombinant LOXL2 proteins [1]. Finally, spectroscopic studies of LOXL2 and LTQ cofactor-derivatized LOXL2 revealed that the structure around the LOXL2 LTQ cofactor differs from the quinone cofactors (if the other copper amine oxidases) in that it is more exposed to solvent than the others [2]. This is likely related to the different inhibitor and substrate specificities of lysyl oxidases compared to all other copper amine oxidases. Taken together, the studies by Rodriguez-Pascual and Minae Mure and colleagues provide a wealth of new information regarding the biosynthesis and molecular features of members of the lysyl oxidase protein family.

Three new reviews, respectively, provide interesting, up-to-date hypotheses and summaries of the roles of lysyl oxidases in cancer, extracellular matrix homeostasis, and remaining questions regarding the biological activities of the lysyl oxidase propeptide and its interesting polymorphic variant [7,10,11].
